# 

**Published:** 1875-04

**Authors:** 


### TABLE OF CONTENTS.

ORIGINAL COMMUNICATIONS.

On the Heredity of Retinitis Pigmentosa. By Dr. Herman Schmidt. ....... 193
Odontolithus. By J. M. Lucas, M.D., Middleport, Ohio. ................. 196
Is the White Blood Corpuscle and the Pus Corpuscle Identical? By T. Curtis Smith, M.D., Middleport Ohio........................................ 198
Death from Encephalic Inflammation Consequent upon Aural Disease. By Swan M. Burnett, M.D., Knoxville, Tenn.............................. 201

SELECTIONS.

Babies' Sore Eyes. By Henry W. Williams, A.M., M.D.... ............... 203
Manual Dilatation of the Os Uteri as a Means of Inducing Premature Labor. By A. D. Sinclair, M.D.......................... ................ 207
Croton-Chloral Hydrate in Megrim. By Sydney Ringer, M.D .............. 213
Pyemia. By E. C. White, of La Grange...... ... ... ............... .... 217
The Action of Phosphorus as a Stimulant. By John Brunton, M.A., M.D... 220
Acute Rheumatism ................................................... 222
Treatment of Erysipelas........ ........ ........................... 223

EXTRACTS AND GLEANINGS.

Gonorrhœa—225. Treatment of Acute and Chronic Bronchitis and Asthma—226. The Wet Sheet in Scarlatina; Soothing Application in Herpes Zoster—227. New Researches on Diabetes; Remedy in Dysmenorrhœa—228. Eczema of the Ear; Onychia Maligna and Ingrowing Nail Fingers—229. Psoriasis Treated by the Internal Use of Carbolic Acid; A Difficulty in Fœtal Auscultation—230. The Elastic Ligature; Suppurating Bubo—231. Dysmenorrhœa; Treatment of Hydrarthrosis—232. Cold-Powder—233. The Capacity of Plants to Absorb Arsenic from the Earth; Pruritus Vaginæ—234. Rheumatism; On Iced Clysters in Dysentery—235. The Treatment of Boils—236. Syphilitic Lichen: The Local Application of Bromine in the Treatment of Carcinoma of the Cervix—237. Bloodless Operations; Silicate of Soda in Gonorrhœa—238. Ergotin in Croupous Pneumonia; Otto of Roses—239. Modern Medical Treatment; Ice in Diphtheria; Chronic Orchitis—240. The Treatment of Ammoniacal Cystitis by Benzoic Acid; Chlorate of Potash in Ozœna; Chloral Subcutaneously in Asthma—241. On Persistent Hemorrhage; Night Sweats of Phthisis; Whooping Cough—242. The Use of Carbolic Acid in Cheesy Pneumonia; Catarrh and Paralysis of the Bladder; Iodide of Potassium in Diphtheria—243. Boldo; Hysterical Spasms; Chronic Ulcers—244. The Stamp Act on Medicines; Acute Articular Rheumatism; Acute Hematuria Treated Successfully by Local Applications—245. A Mode of Reducing Strangulated Hernia; Aromatic Syrup of Senna; Diarrhœa of Phthisis—346. The Preservation of Leeches; Treatment of Pleurisy; Sprains—247. Rheumatism and Neuralgia; Phagedena; Treatment of Hemorrhoids by Injections of Ergot—248. Preservation of Vegetable Infusicn by Chloroform; A Diagnostic Hint in old Ulcers; Compound Tincture of Opium—249. New Process for Rendering Wood Incombustible; Tonic Prescription; Dysentery; Menorrhagia—250. The Action of Morphia Subcutaneously Injected; Treatment of Typhoid Fever by Air Baths; Arsenic in Asthma; Sulpho-Carbolate of Zinc in Pruritis—251. The Artichoke in Rheumatism; Treatment of Spasmodic Asthma; Gonorrhœa; Indurated Bubo; Mammary Abscesses—252.

EDITORIAL AND MISCELLANEOUS.

Action of the Academy of Medicine. .............. ................... 253
A Card; Extract............. ....................................... 254
List of Cash Payments to the Record.................................. 255

